# Cyclophilin D Is Involved in the Regulation of Autophagy and Affects the Lifespan of *P. anserina* in Response to Mitochondrial Oxidative Stress

**DOI:** 10.3389/fgene.2016.00165

**Published:** 2016-09-14

**Authors:** Piet Kramer, Alexander T. Jung, Andrea Hamann, Heinz D. Osiewacz

**Affiliations:** Department of Biosciences, Molecular Developmental Biology, Institute of Molecular Biosciences and Cluster of Excellence Frankfurt Macromolecular Complexes, J. W. Goethe UniversityFrankfurt, Germany

**Keywords:** cyclophilin D, autophagy, lifespan, aging, mitohormesis, oxidative stress

## Abstract

The mitochondrial permeability transition pore plays a key role in programmed cell death and the induction of autophagy. Opening of the pore is regulated by the mitochondrial peptidyl prolyl-cis, trans-isomerase cyclophilin D (CYPD). Previously it was shown in the aging model organism *Podospora anserina* that PaCYPD abundance increases during aging and that *PaCypD* overexpressors are characterized by accelerated aging. Here, we describe a role of PaCYPD in the regulation of autophagy. We found that the accelerated aging phenotype observed in a strain overexpressing *PaCypD* is not metacaspase-dependent but is accompanied by an increase of general autophagy and mitophagy, the selective autophagic degradation of mitochondria. It thus is linked to what has been defined as “autophagic cell death” or “type II” programmed cell death. Moreover, we found that the previously demonstrated age-related induction of autophagy in wild-type aging depends on the presence of PaCYPD. Deletion of *PaCypD* leads to a decrease in autophagy in later stages of age and under paraquat-mediated oxidative stress. Finally, we report that PaCYPD is also required for mitohormesis, the beneficial effect of mild mitochondrial stress. Thus, PaCYPD plays a key role in the context-dependent regulation of pathways leading to pro-survival and pro-death effects of autophagy.

## Introduction

Programmed cell death (PCD) is a cell-suicide program that is essential for proper development but is also involved in degenerative processes leading to aging and to various diseases. Due to the morphology of dying cells this process is classified into three main types: apoptotic or “type I,” autophagic or “type II,” and regulated necrotic or “type III” PCD (Schweichel and Merker, 1973; Kroemer et al., 2005). According to the Nomenclature Committee on Cell Death, extrinsic apoptosis strictly culminates in a caspase-dependent cell death, while intrinsic apoptosis leads to the release of pro-apoptotic factors from the mitochondrial intermembrane space. The latter is further subdivided into a caspase-dependent and caspase-independent cell death based on its dependency on “apoptosis-inducing factors” (AIFs) or the activation of caspases. In contrast, autophagic cell death is accompanied by an increased autophagic flux and can be blocked via the inhibition of autophagy. Despite its role in PCD, under physiologic conditions, autophagy acts as a pro-survival mechanism involved in metabolic adaptation and the recycling of damaged or surplus cellular components. The third type of PCD, termed regulated necrosis, is defined as a genetically controlled cell death process that lacks the classical markers of apoptosis and autophagy and often involves the activation of the kinases receptor-interacting protein 1 (RIP1) and its homolog RIP3 (Galluzzi et al., 2012).

A key process preceding the induction of autophagy and PCD is the permeability transition of the mitochondrial inner membrane resulting from an opening of the mitochondrial permeability transition pore (mPTP). Regulation of the mPTP is mediated by the mitochondrial peptidyl-prolyl cis, trans isomerase cyclophilin D (CYPD; Baines et al., 2005; Basso et al., 2005; Nakagawa et al., 2005; Schinzel et al., 2005). Oxidative stress and elevated Ca^2+^-levels lead to binding of CYPD to and opening of the mPTP. The resulting influx of water into mitochondria leads to dissipation of the mitochondrial membrane potential, swelling of the organelle, unfolding of the inner membrane, rupture of the outer membrane, release of apoptogens, and the induction of PCD via regulated apoptosis or necrosis (Schneider, 2005; Bonora et al., 2015; Izzo et al., 2016). A role of the mPTP in the degradation of mitochondrial proteins by autophagy has been reported in mammals (Carreira et al., 2010). Starvation of cardiac cells was found to result in a reduction of the mitochondrial membrane potential and the degradation of mitochondrial proteins by autophagy, which both could be prevented by treatment with the mPTP-inhibitor cyclosporine A (CsA). Furthermore, cardiomyocytes from mice overexpressing *CypD* exhibited an increased level of autophagy even under fed conditions.

In the fungal aging model *Podospora anserina* first evidence for a role of PaCYPD in aging is derived from a mitochondrial proteome analysis that revealed an age-related increase of PaCYPD (Groebe et al., 2007). Subsequent investigations showed that *PaCypD* deletion mutants display an increased resistance against inducers of oxidative stress and cell death. In addition, *PaCypD* overexpressing strains were characterized by premature nuclear condensation, cytochrome c release, reduction of the membrane potential, a severe reorganization of the mitochondrial ultrastructure, and a strong reduction of the lifespan (Brust et al., 2010a). In contrast to the role of PCD in higher eukaryotes were PCD is involved in the control of cellular homeostasis and the survival of the organism, both in the yeast *Saccharomyces cerevisiae* and in *P. anserina* apoptosis acts in the final step in the life cycle leading to death of the individual (Hamann et al., 2008). The latter is demonstrated by the finding that ablation of the two metacaspases of *P. anserina* and of “AIFs” lead to increased tolerance to oxidative stress and an increased lifespan. Since these data are consistent with an age-related increase of the metacaspase activity in the wild type, it was suggested that apoptosis is induced by an increase of oxidative stress in the senescent state (Hamann et al., 2007; Brust et al., 2010b). More recent data identified also a role of autophagy in aging of *P. anserina* (Philipp et al., 2013; Knuppertz et al., 2014) and now raise the question about mechanistic links between autophagy, CYPD, mPTP, and lifespan control.

Here we report data from a study analyzing the role of the two metacaspases and of autophagy in *PaCypD* overexpressing strains of *P. anserina*. We show that *PaCypD* overexpressors are characterized by an increased autophagy-dependent degradation of mitochondrial and cytosolic proteins and that this process, and not the induction of a metacaspase-dependent PCD (“type I” PCD), is responsible for the reduced lifespan of *PaCypD* overexpressing strains. In contrast, during physiological (“normal”) aging of the wild type, PaCYPD mediates a moderate induction of autophagy in response to oxidative stress and aging, which acts as a pro-survival pathway.

## Materials and methods

### Strains and culture conditions

The following *P. anserina* strains were used: Wild-type strain “s” (Rizet, 1953), the *PaCypD*-mutants Δ*PaCypD, PaCypD_OEx* (Brust et al., 2010a), the metacaspase deletion mutants Δ*PaMca1*, Δ*PaMca2*, Δ*PaMca1/* Δ*PaMca2* (Hamann et al., 2007), the autophagy-deficiency mutant Δ*PaAtg1* (Knuppertz et al., 2014), the *PaSod3*-overexpressing mutant *PaSod3_OEx* and the previously described mutants *PaSod1::Gfp, PaSod3::Gfp* (Zintel et al., 2010). These strains were used for the generation of the new mutants Δ*PaCypD/PaSod3::Gfp, PaCypD_OEx/PaSod3::Gfp*, Δ*PaCypD/ PaSod1::Gfp, PaCypD_OEx/PaSod1::Gfp* in addition to *PaCypD_OEx/* Δ*PaAtg1* and *PaCypD_OEx/*Δ*PaMca1/* Δ*PaMca2*, which were verified by Southern blot and western blot analysis (Supplementary Figures 1–8). All strains were constructed in the genetic background of the wild-type strain “s.” Strains were grown on complete medium (BMM) containing cornmeal extract (Esser, 1974) at 27°C under constant light. For spore germination, the BMM medium was supplemented with 60 mM ammonium acetate and spores were incubated at 27°C in the dark for 2 d. All used strains originated from monokaryotic ascospores isolated from irregular asci. For subsequent experiments strains were either grown on solid semi-synthetic M2 medium (M2) or in liquid complete medium (CM) at 27° under constant light (Osiewacz et al., 2013).

### Southern blot analysis

Total DNA was isolated according to standard protocols (Lecellier and Silar, 1994). DNA restriction, gel electrophoresis, and Southern blotting were performed according to standard protocols (Sambrook et al., 1989). As a hybridization probe specific for the phleomycin resistance gene (*Ble*), the 1293 bp BamHI-fragment (ER0051, Thermo Scientific) of the plasmid pKO4 (Hamann et al., 2005; Luce and Osiewacz, 2009) was used. The 727 bp NcoI/ClaI-fragment (ER0571, ER0141, Thermo Scientific) of the plasmid pKO7 (Kunstmann and Osiewacz, 2009) was used as a hybridization probe specific for the hygromycin resistance gene (*Hph*). For Southern blot hybridization and detection of hybridizing bands, the instructions of the supplier (Roche, Germany) were followed.

### Lifespan and growth rate determination

The lifespan and growth rate of monokaryotic isolates was determined using race tubes containing M2 medium as described (Kunstmann and Osiewacz, 2008). The lifespan of *P. anserina* is defined as the time period in days (d) of linear hyphal growth whereas the growth rate is defined as the measured growth in centimeters (cm) per time period in days (d). For the analysis of the growth rate and lifespan the growth front was marked every 1–3 days until death of the individuals. From these data the mean lifespan was calculated as average of all individual isolates from each strain as previously described (Osiewacz et al., 2013). To determine the lifespan under oxidative stress or in presence of cyclosporine A, petri dishes containing 30 ml M2 medium with 80 or 160 μM of paraquat (Sigma-Aldrich, 856177), CuSO_4_, a combination of both or 0.05 μg/ml CsA were inoculated with mycelium from germinated spores. Plates were incubated at 27°C under constant light or in the dark in case of plates containing light sensitive cyclosporine A.

### Measurement of metacaspase activity

For the generation of extracts of 11 days old cultures mycelium from 2 days old germinated spores of four different isolates of each strain (= biological replicates) were grown on M2 medium for 5 days at 27°C under constant light. M2 medium petri dishes covered with cellophane were inoculated with pieces of mycelium that derived from the growth front and incubated for additional 2 days. Afterwards the grown mycelia was transferred into CM-liquid medium and incubated for 2 further days at 27°C under constant light and shaking at 180 rpm. The harvested mycelia were ground in liquid nitrogen and used for the isolation of total protein extracts and measurement of the arginine-specific peptidase activity using the fluorochrome-coupled peptide Z-GGR-Amc as a substrate as described (Hamann et al., 2007). Peptidase activity in total protein extracts from each strain was measured twice.

### Western blot analysis

Cultivation of at least three different isolates of each *P. anserina* strain was carried out as described above for the determination of the metacaspase activity. For the cultivation of 6 days old strains M2 medium petri dishes covered with cellophane were directly inoculated with mycelium from germinated spores. In case of growth under oxidative stress 60 μM paraquat (Sigma-Aldrich, 856177) was added to CM liquid medium. Harvested mycelia were ground in liquid nitrogen and used for the isolation of total protein extracts with 1:100 protease inhibitor cocktail set IV (Calbiochem) as described (Osiewacz et al., 2013). One hundred micrograms total protein extracts were fractionated by 2-phase SDS-PAGE (12% separating gels) according to the standard protocol (Brust et al., 2010a). After electrophoresis, proteins were transferred to PVDF membranes (Millipore, IPFL00010). Blocking, antibody incubation, and washing steps were performed according to the Odyssey western blot analysis handbook (LI-COR Biosciences, Bad Homburg, Germany). The following primary antibodies were used: Anti-GFP (mouse, 1:10,000 dilution, Sigma-Aldrich, G6795), anti-PaCYPD (rabbit, 1:5000 dilution) previously described in Brust et al. (2010a). In all analyses, secondary antibodies conjugated with IRDye680RD (1:15,000 dilution, goat anti-mouse: LI-COR Biosciences, 926–68,070) or IRDye800CW (1:15,000 dilution, goat anti-rabbit: LI-COR Biosciences, 926–32,211) were used. The Odyssey infrared scanner (LI-COR Biosciences) was used for detection and quantification using the manufacturer's software.

### Statistical analysis

For statistical analyses of growth rate, metacaspase activity, and quantification of the GFP protein level in western blot analyses the 2-tailed student's *t*-test was used. For statistical analysis of lifespan the 2-tailed Wilcoxon rank-sum test was used. For all analyses statistical significance was calculated and defined as not significant (*P* > 0.05); as significant (^*^P < 0.05); as highly significant (^**^P < 0.01); very highly significant (^***^P < 0.001).

## Results

### Lifespan reduction in *PaCypD* overexpressors does not rely on metacaspase induction

**Figure 1:**
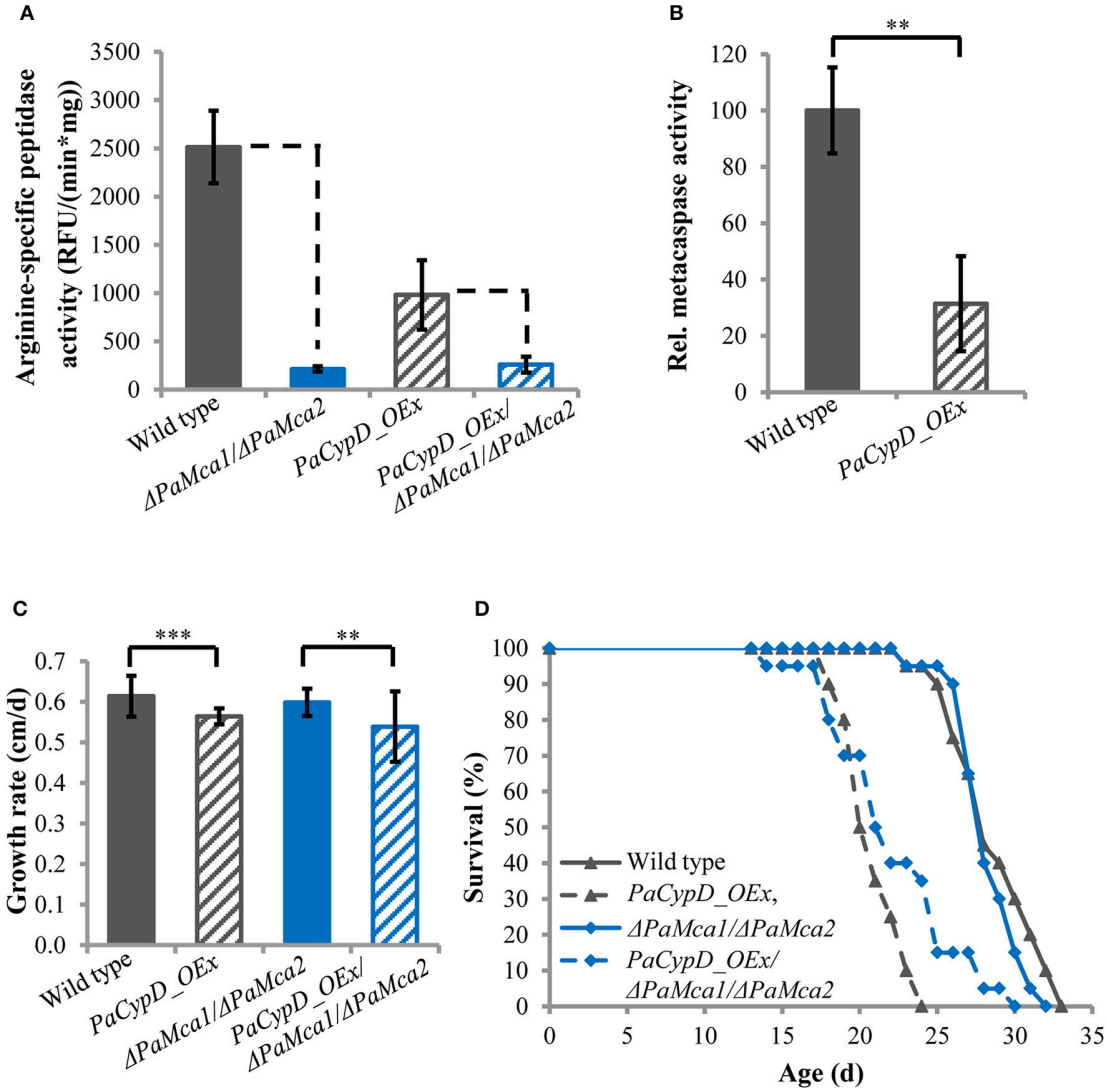
**(A)** Arginine-specific peptidase activity [relative fluorescence units (RFU)/(min^*^mg)] in total protein extracts of mycelium of 11 d old wild type, *PaCypD_OEx* and the different deletion strains. As substrate, the fluorochrome-coupled peptide Z-GGR-Amc was used. Fifty micrograms total protein extracts of four different isolates of each strain were measured. **(B)** Relative metacaspase activity was determined as difference of the arginine-specific peptidase activity from Panel **A** between wild type and Δ*PaMca1/*Δ*PaMca2* or *PaCypD_OEx* and *PaCypD_OEx/*Δ*PaMca1/*Δ*PaMca2*. The mean metacaspase activity of the wild type was set to 100%. **(C)** Growth rate and **(D)** lifespan of 20 different isolates of the wild type, *PaCypD_OEx*, Δ*PaMca1/*Δ*PaMca2*, and the triple mutant *PaCypD_OEx/*Δ*PaMca1/*Δ*PaMca2* on M2 medium. Data represent average ± SEM (2-tailed student's *t*-test), ^**^P < 0.01, ^***^P < 0.001.

Previously it was shown that PaCYPD is involved in the induction of PCD. The analysis of a *PaCypD-*overexpressing strain with an ectopic integration of the plasmid pCypDEx1 containing *PaCypD* under the control of the constitutive promoter of the metallothionein gene *PaMt1*, identified an accelerated cytochrome c release, nuclear condensation and a decrease in membrane potential as markers for an apoptotic type of PCD (Brust et al., 2010a). However, the study did not address the dependence of the underlying processes on the two *P. anserina* metacaspases PaMCA1 and PaMCA2 and thus not explicitly demonstrated a metacaspase-dependent ‘type I’ PCD. In order to investigate this role, we constructed a *PaCypD_OEx/*Δ*PaMca1/*Δ*PaMca2* triple mutant in which both *PaMca* genes are deleted in the *PaCypD* overexpression background. Subsequently, we determined the arginine-specific peptidase activity of 11 days old cultures of the wild type, the *PaCypD* overexpressor, and the *PaMca1/PaMca2* double deletion in the wild-type and the *PaCypD* overexpression background (Figure 1A). The arginine-specific peptidase activity of the Δ*PaMca1/*Δ*PaMca2* deletion strain is very low [214 ± 27 RFU/(min^*^mg)] compared to the activity of the wild type [2514 ± 376 RFU/(min^*^mg)] indicating that the activity mainly results from the activity of PaMCA1 and PaMCA2. Strikingly, the *PaCypD_OEx* mutant is characterized by a considerably lower arginine-specific activity [981 ± 359 RFU/(min^*^mg)] than the wild type, while the activity of the *PaCypD_OEx/*Δ*PaMca1/*Δ*PaMca2* triple mutant [258 ± 82 RFU/(min^*^mg)] is comparable to that of the Δ*PaMca1/*Δ*PaMca2* deletion strain. From these data the metacaspase activity (Figure 1B) was calculated as the difference in the arginine-specific peptidase activity between wild type and Δ*PaMca1/*Δ*PaMca2* strain or *PaCypD_OEx* and *PaCypD_OEx/*Δ*PaMca1/*Δ*PaMca2* triple mutant. Surprisingly, compared to the wild type of the same age, the metacaspase activity in 11 days old *PaCypD* overexpressors is strongly reduced.

Next we determined the growth rate (Figure 1C) and lifespan (Figure 1D) of the different strains on semi-synthetic M2 medium. On this medium the growth rate and mean lifespan (Supplementary Figure 9) of the wild type and the Δ*PaMca1/*Δ*PaMca2* strain do not significantly differ from each other. In contrast, overexpression of *PaCypD* in the wild-type and the Δ*PaMca1/*Δ*PaMca2* genetic background leads to a similar reduction of the growth rate and the mean lifespan. Overall these data demonstrate that the accelerated aging process of *PaCypD* overexpressors does not rely on metacaspase-activity and “type I” PCD.

### Lifespan reduction in *PaCypD* overexpressing strains depends on *PaCypD*-mediated induction of autophagy

**Figure 2:**
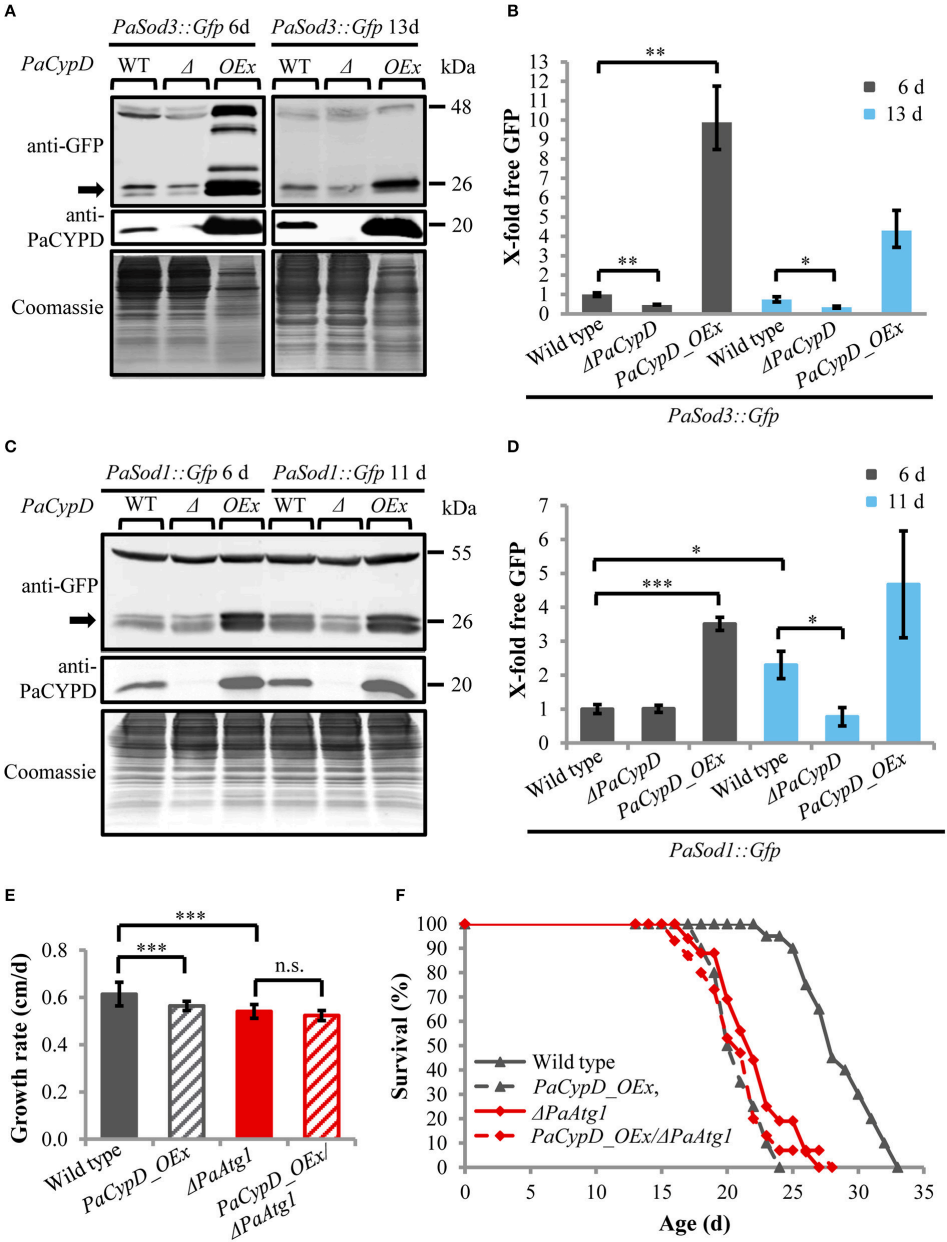
**Autophagy-dependent degradation of mitochondrial and cytosolic marker proteins in wild-type strain and different ***PaCypD*** mutants during aging. (A)** Monitoring mitophagy by western blot analysis using the mitochondrial protein PaSOD3::GFP. 6 and 13 d old wild-type (WT), Δ*PaCypD* and *PaCypD_OEx* strains expressing *PaSod3::Gfp* were cultured for 2 d in CM medium. “Free GFP” (indicated by arrow) and PaCYPD were monitored by immunoblotting with anti-GFP and anti-PaCYPD in 100 μg total protein extracts. The positions of molecular mass markers are indicated on the right. **(B)** The GFP protein levels of three different isolates of each strain were quantified and normalized to the Coomassie-stained gels (loading control). The protein amount present in the 6 d old wild type was set to one. **(C)** Monitoring autophagy as described in **(A)** except using 6 and 11 d old strains expressing *PaSod1::Gfp*. **(D)** Quantification of “free GFP” derived from the cytosolic marker PaSOD1::GFP as described in **(B)**. **(E)** Growth rate and **(F)** lifespan of wild type (*n* = 20), *PaCypD_OEx* (*n* = 20), Δ*PaAtg1* (*n* = 16), and the double mutant *PaCypD_OEx/*Δ*PaAtg1* (*n* = 15) on M2 medium. Data represent average ± SEM (2-tailed student's *t*-test), ^*^P < 0.05, ^**^P < 0.01, ^***^P < 0.001.

Next we addressed a link of PaCYPD and autophagy. For the demonstration of the vacuolar degradation of mitochondrial and cytosolic proteins via mitophagy and general autophagy, we utilized a recently established modified assay (Knuppertz et al., 2014) that originally was developed for the yeast *S. cerevisiae* (Meiling-Wesse et al., 2002; Welter et al., 2010). In this assay the degradation of reporter proteins is analyzed. In our analysis, mitochondrial PaSOD3::GFP served as a marker for mitophagy and cytosolic PaSOD1::GFP for general autophagy. After autophagy-mediated degradation of parts of the fusion proteins in the vacuole, “free GFP” remains stable and can be quantified by western blot analysis. We analyzed “free GFP” in total protein extracts of two different age stages of the wild type, the *PaCypD* overexpressor and the *PaCypD* deletion strain, which was obtained by homologous replacement of *PaCypD* by a selection marker containing a blasticidin resistance (*Bsd*) and phleomycin resistance (*Ble*) gene for selection in *Escherichia coli* and *P. anserina* (Brust et al., 2010a; Figures 2A,C). Compared to the wild type, the amount of “free GFP” derived from the PaSOD3::GFP mitophagy reporter protein was significantly lower in total protein extracts of Δ*PaCypD* of both 6 and 13 days old cultures. In striking contrast, compared to 6 days old cultures of the wild type, a 10-fold increase of “free GFP” was detected in extracts of *PaCypD* overexpressors of the same chronological age. Also, at the age of 13 days *PaCypD* overexpressing strains exhibit a higher amount of “free GFP” (Figure 2B). In addition, the analysis of “free GFP” resulting from degradation of the general autophagy reporter PaSOD1::GFP revealed that 6 days old wild-type and Δ*PaCypD* cultures display equal amounts of “free GFP,” indicating that the basal level of general autophagy is independent of PaCYPD. In striking contrast, we verified the age-related increase in general autophagy first reported for the wild type of *P. anserina* (Knuppertz et al., 2014) and found that it is PaCYPD-dependent. In Δ*PaCypD* strains “free GFP” of 11 days old cultures is significantly lower than in wild-type strains of the same age and no significant difference of “free GFP” is observed in 6 and 11 days old Δ*PaCypD* cultures. In addition, compared to 6 days old wild-type cultures, *PaCypD* overexpressors display a 3.5-fold increase of “free GFP” in 6 days cultures and a 4.6-fold increase at an age of 11 days (Figure 2D). The mitophagy reporter PaSOD3::GFP may also be degraded (together with cytosolic compounds) unspecifically to some extent in the *PaCypD* overexpressor. However, since the increase in degradation of PaSOD3::GFP is much higher than the increase in PaSOD1::GFP degradation (10-fold vs. 3.5-fold) a preferential degradation of PaSOD3::GFP via mitophagy can be concluded.

Next we investigated the impact of autophagy on growth and lifespan of *PaCypD* overexpressors. Ablation of PaATG1, an essential component of the autophagic machinery controlling early steps in autophagosome formation, impairs the pro-survival function of autophagy and results in a reduction of the growth rate and a short-lived phenotype (Knuppertz et al., 2014). Most strikingly, we found that growth rate and lifespan of the *PaCypD_OEx/*Δ*PaAtg1* double mutant is not further reduced in comparison to the two single mutants (Figures 2E,F, Supplementary Figure 10), indicating an epistatic relation of *PaAtg1* and *PaCypD*. Thus, a pro-survival role of autophagy, as it is found in the wild type, can be excluded in the *PaCypD* overexpressor. We conclude that lifespan reduction in this mutant is caused by the CYPD-dependent strong induction of general autophagy and mitophagy. PaCYPD thus appears to be involved in the switch from a pro-survival to a pro-death role of autophagy. Moreover, as indicated by the lifespan reduction in the *PaCypD_OEx/*Δ*PaAtg1* double mutant, a complete suppression of autophagy in the *PaCypD* overexpression background is also not beneficial clearly demonstrating that balancing of autophagy flux is important for cellular homeostasis.

### PaCYPD is involved in paraquat-mediated ROS signaling

**Figure 3:**
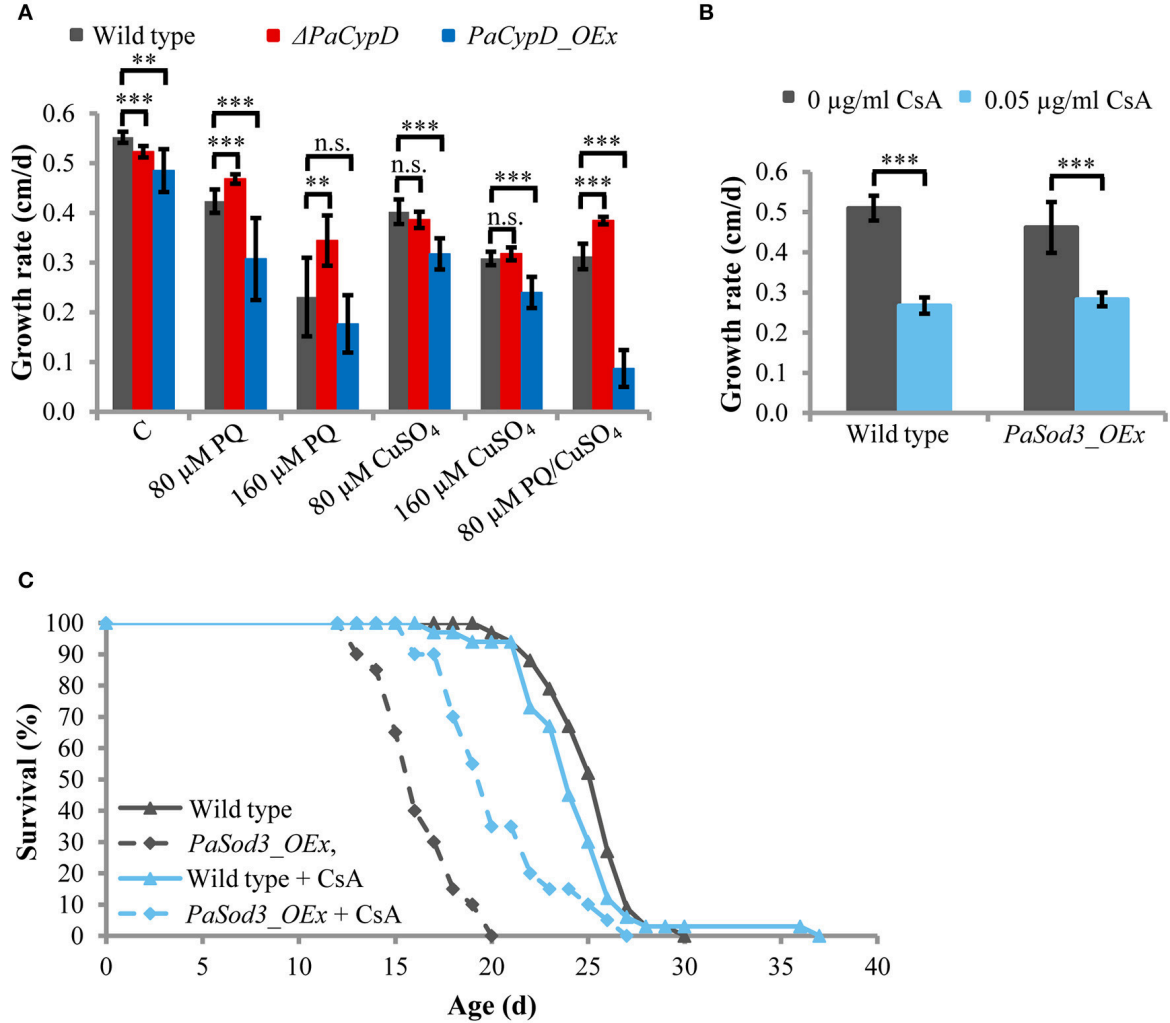
**(A)** The growth rate of 2 d old wild-type, Δ*PaCypD* and *PaCypD_OEx* strains (*n* = 10) was monitored for 13 d on M2 medium in the presence of the ROS stressors paraquat (PQ), CuSO_4_, both or without ROS stressors (control: C). **(B)** Growth rate and **(C)** lifespan of wild type (*n* = 33) and *PaSod3_OEx* (*n* = 20) on M2 medium with 0 or 0.05 μg/ml cyclosporine A. Data represent average ± SEM (2-tailed student's *t*-test). Not significant (n.s.), ^**^P < 0.01, ^***^P < 0.001.

After the identification of a role of PaCYPD in the induction of autophagy we set out to investigate the underlying mechanisms in more detail. Since a role of ROS in aging and in the regulation of CYPD-induced mPT is well documented (Linard et al., 2009; Davalli et al., 2016), we next analyzed the impact of oxidative stress on the growth rate of the wild type and different *PaCypD* mutants (Figure 3A). We induced global oxidative stress in all cellular compartments via the addition of CuSO_4_ to the growth medium. In addition, the herbicide paraquat was used to induce mitochondrial stress. Under standard conditions without the application of the two stressors, the growth rate of Δ*PaCypD* and *PaCypD_OEx* strains is slightly reduced compared to the growth rate of the wild type. On medium containing 80 and 160 μM CuSO_4_, respectively, the growth rate of Δ*PaCypD* and the wild type do not differ. Growth of *PaCypD_OEx* is only slightly decreased compared to the wild type, indicating that the tolerance to Cu^2+^-induced global cellular oxidative stress is independent from PaCYPD. In contrast, compared to the wild type, 80 and 160 μM paraquat in the growth medium resulted in a significant increase of the growth rate of Δ*PaCypD* and a decreased growth of *PaCypD_OEx* on medium containing 80 μM paraquat. It appears, that Δ*PaCypD* strains are more tolerant to paraquat-mediated mitochondrial ROS stress. While all three investigated strains were able to grow on 160 μM CuSO_4_ or 160 μM paraquat, respectively, only the wild type and the *PaCypD* deletion strain were tolerant to the addition of 80 μM of both ROS stressors. Under these conditions, growth of *PaCypD_OEx* was almost completely inhibited. These data are consistent with observations in higher eukaryotes in which CYPD facilitates ROS-induced mPTP-opening leading to the induction of cell death.

To validate this latter role in *P. anserina*, we tested whether the reduced lifespan of a mutant overexpressing *PaSod3*, encoding the mitochondrial superoxide dismutase, can be restored by the CYPD-inhibitor cyclosporine A. The mutant is characterized by increased ROS stress (Grimm and Osiewacz, 2015). Upon application of 0.05 μg/ml cyclosporine A to the growth medium, the growth rates of the wild type and *PaSod3_OEx* are significantly decreased, but do not differ from each other (Figure 3B). The cyclosporine A-mediated impairment of the growth rate is consistent with earlier findings reported for *Neurospora crassa* (Tropschug et al., 1989; Bardiya and Shiu, 2007) and was assumed to result from a PaCYPD/CsA interaction product, since the growth rate of Δ*PaCypD* is unaffected by CsA (Brust et al., 2010a). Moreover, chronic treatment with cyclosporine A does not affect the mean lifespan of the wild type. In striking contrast, the lifespan of *PaSod3_OEx* is significantly increased from 16.3 ± 2 to 20.4 ± 3 days (Figure 3C, Supplementary Figure 11). Together these data suggest that PaCYPD-inhibition or deficiency mediates an increased tolerance to mitochondrial but not to global oxidative stress. In addition, premature death caused by high mitochondrial oxidative stress can be counteracted by the CYPD-inhibitor cyclosporine A.

### Under physiological conditions ROS-mediated autophagy is PaCYPD-dependent and acts as a pro-survival pathway

**Figure 4:**
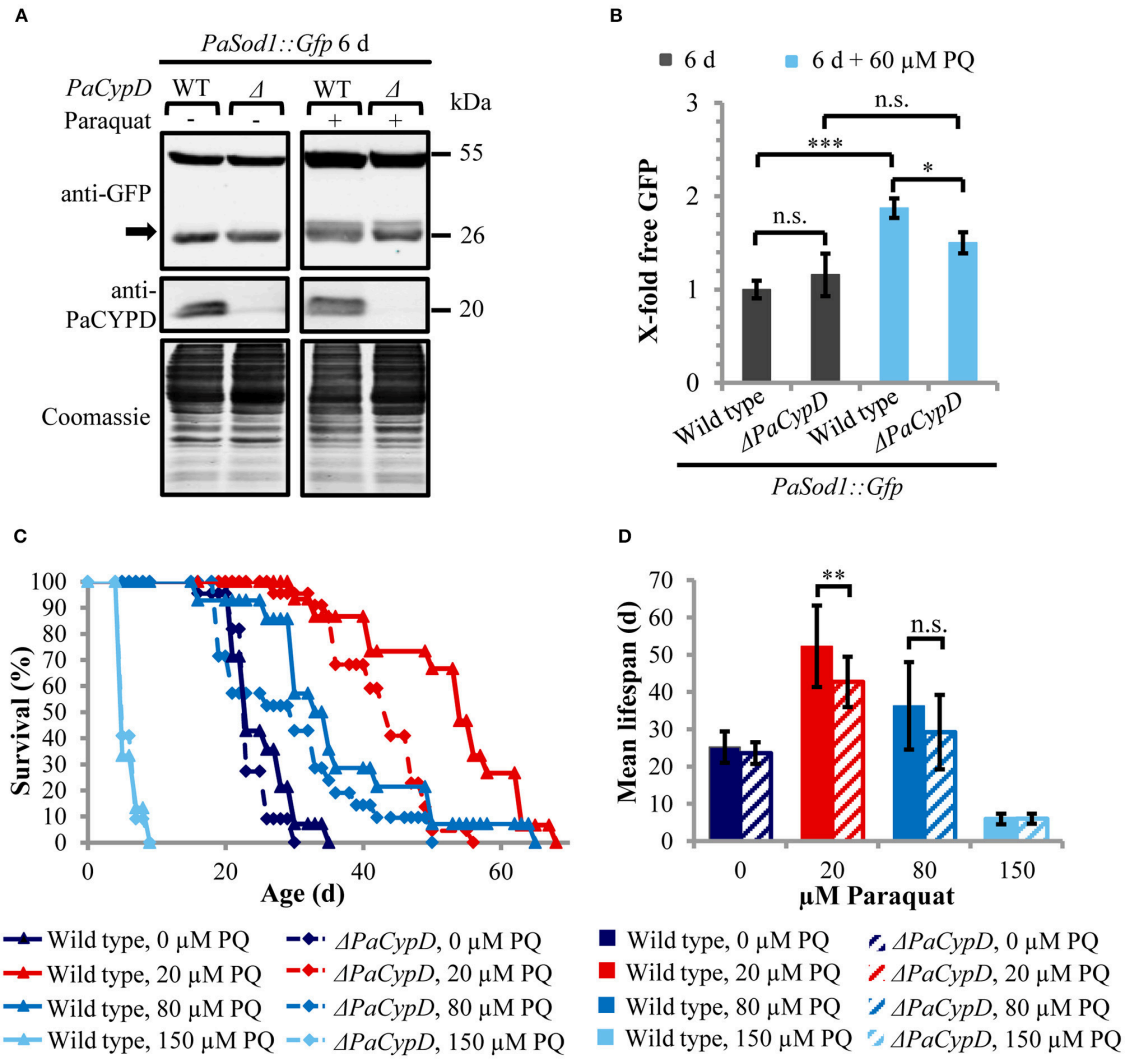
**PaCYPD mediates autophagy and participates in longevity-assurance under ROS stress. (A)** Monitoring autophagy by western blot analysis using the cytosolic protein PaSOD1::GFP. 6 d old wild-type (WT) and Δ*PaCypD* strains expressing *PaSod1::Gfp* were cultured for 2 d in CM medium with or without 60 μM paraquat (PQ). “Free GFP” (indicated by arrow) and PaCYPD was detected by immunoblotting with anti-GFP and anti-PaCYPD in 100 μg total protein extracts. The positions of molecular mass markers are indicated on the right. **(B)** The GFP protein levels of three different isolates of each strain were normalized to the Coomassie-stained gels (loading control) and the protein amount present in the 6 d old wild type was set to one. Data represent average ± SEM (2-tailed student's *t*-test), not significant (n.s.), ^*^P < 0.05, ^***^P < 0.001. **(C)** Lifespan and **(D)** mean lifespan of at least 14 different isolates of the wild-type strain and at least 21 different isolates of the Δ*PaCypD* strains on M2 medium in the presence of 0, 20, 80, or 150 μM paraquat (PQ). Data represent average ± SEM (2-tailed Wilcoxon rank-sum test) Not significant (n.s.), ^**^P < 0.01. At note: Survival curves of wild type and Δ*PaCypD* in presence of 150 μM PQ are largely overlapping and therefore difficult to distinguish.

Since CYPD-mediated opening of the mPTP can be induced by ROS and since we recently found that autophagy is also induced by ROS in *P. anserina* (Knuppertz et al., submitted), we next analyzed whether the induction of autophagy depends on PaCYPD. In this study we compared “free GFP” levels in 6 days old cultures of wild-type and Δ*PaCypD* strains challenged by 60 μM paraquat (Figure 4A). As a control we used the strains of the same age grown in medium without paraquat from the age-related study described above (Figure 2D). In the wild type, paraquat led to a two-fold increase of “free GFP” (Figure 4B). Although paraquat also led to increased “free GFP” in Δ*PaCypD*, the level is significantly lower than in the wild type indicating that the paraquat-mediated induction of autophagy is indeed, at least partially, dependent on *PaCypD*.

Next we investigated whether the beneficial autophagy-dependent effect of mild paraquat stress identified in a parallel study (Knuppertz et al., submitted) also depends on PaCYPD. We compared the lifespans of the wild type and the Δ*PaCypD* mutant grown on standard growth medium containing increasing amounts of paraquat (Figure 4C). Strikingly, we found that the lifespan-extending effect of paraquat observed for the wild type is strongly reduced in the Δ*PaCypD* strain. On medium containing 20 μM paraquat the difference in mean lifespan is significant (Figure 4D). In contrast, at 80 μM the difference in mean lifespan is not significant and under harsh paraquat stress (i.e., 150 μM) the lifespan is strongly reduced in both strains. These results are consistent with previous investigations in other organisms like *S. cerevisiae* or *Caenorhabditis elegans* (Cypser and Johnson, 2002; Mesquita et al., 2010; Yang and Hekimi, 2010) and provide mechanistic clues explaining the beneficial effect of mild stress via the induction of adaptive responses leading to increased stress tolerance. They support a beneficial role of PaCYPD in the mediation of mitohormesis.

## Discussion

Previous work identified PCD as the final step in the lifetime of the fungal aging model *P. anserina*. Different components of a PCD machinery including “apoptosis inducing factors” (AIFs), poly(ADP-ribose) polymerase (PARP), metacaspases (MCAs), and the mitochondrial peptidyl prolyl-cis, trans-isomerase cyclophilin D (CYPD) were found to be involved in PCD and impact *P. anserina* lifespan (Groebe et al., 2007; Hamann et al., 2007; Brust et al., 2010a,b; Müller-Ohldach et al., 2011; Daum et al., 2013; Strobel and Osiewacz, 2013). In the current study, we report a role of one of these components, PaCYPD, in the induction of autophagy, a pathway that previously was identified as a pro-survival mechanism involved in longevity assurance in *P. anserina* (Philipp et al., 2013; Knuppertz et al., 2014).

We started our study with the investigation of a short-lived mutant in which *PaCypD* is strongly overexpressed. The mutant was previously shown to be severely impaired in mitochondrial ultrastructure, membrane potential and other characteristics of induced PCD (Brust et al., 2010a). We now demonstrate that in this mutant accelerated aging and PCD is metacaspase-independent and is characterized by a strong induction of mitophagy and general autophagy. In addition, we observed that the strong increase in autophagic flux does not function as a pro-survival mechanism but is rather the cause of the deleterious effects of *PaCypD* overexpression. Death in this mutant thus confers to what has previously been defined as “type II” PCD or “autophagic cell death” and general autophagy and mitophagy represent pro-death pathways. Our data about a role of PaCYPD and the mPTP in the induction of autophagy are well consistent with what has been previously reported in mammals. For instance, in rat hepatocytes serum deprivation and glucagon caused the depolarization of mitochondria, followed by the sequestration of these mitochondria by autophagosomes, a process that can be prevented by the CYPD-inhibitor CsA (Elmore et al., 2001; Rodriguez-Enriquez et al., 2006). In addition, it was shown in myocytes from mice overexpressing *CypD* that autophagy is enhanced even under fed (non-starvation) conditions (Carreira et al., 2010). The pro-death role of PaCYPD-mediated mitochondrial permeability transition under severe stress is further emphasized by the lifespan-extending effect of the CYPD-inhibitor CsA in a *P. anserina* mutant overexpressing *PaSod3*, which suffers from high oxidative stress and is also short-lived (Zintel et al., 2010; Grimm and Osiewacz, 2015). Although it is currently not explicitly shown whether death of this mutant is mediated by an autophagic or apoptotic type of PCD, the characteristics of the mutant suggest that it is likely that PaCYPD-mediated “type II” PCD is executed in this mutant.

Apart from the role of PaCYPD and autophagy in situations of severe stress our current study also casts new light on the role and regulation of autophagy during physiologic (“normal”) aging of the wild type and in situations of mild oxidative stress. In particular, we found that the age-related increase in general autophagy first reported for the wild type of *P. anserina* (Knuppertz et al., 2014) is impaired in *PaCypD*-deficient strains. Moreover, compared to the wild type, Δ*PaCypD* strains exhibit a reduction of general autophagy in response to paraquat-mediated oxidative stress. Since superoxide is known to be required for starvation-induced autophagy (Chen et al., 2009) these results are well consistent with the observation that starvation of mice failed to induce autophagy in *CypD*-deficient cardiomyocytes (Carreira et al., 2010). Accordingly, in a *P. anserina* mutant lacking the mitochondrial superoxide dismutase PaSOD3, mitophagy is strongly induced. This induction was concluded to result from the increased mitochondrial superoxide load since treatment with paraquat was shown to induce both autophagy and mitophagy (Knuppertz et al., submitted). In *P. anserina* we obtained first evidence about a role of PaCYPD in pro-survival signaling as a response to mild oxidative stress and its impact on longevity. Although such a hormetic response is known to be triggered by many compounds like rapamycin, spermidine, and resveratrol (Rubinsztein et al., 2011) which are involved in autophagy induction, it is important to consider that hormesis has a pleiotropic basis and is not restricted to autophagy. Moreover, the role of PaCYPD in hormesis is very complex since the physiological role of PaCYPD has been implicated in the regulation of Ca^2+^-homeostasis and mitochondrial metabolism (Elrod and Molkentin, 2013).

In mammals, a role of CYPD in pro-survival and pro-death pathways has been implicated in tissue damage resulting from ischemia-reperfusion injury (IRI), the damage caused by reperfusion of tissue following a period of restricted blood supply (ischemia). Induction of PCD mediated by opening of the mPTP (high-conductance state) is a critical determinant contributing to IRI and can be counteracted by treatment with CsA or genetic ablation of CYPD (Baines et al., 2005). In addition, one of the most protective mechanisms against IRI is ischemic preconditioning (IPC). IPC is pro-survival signaling elicited by transient, reciprocal episodes of ischemia/reperfusion prior to a major ischemic event and represents a special form of hormesis (Zhao et al., 2013). It was shown that IPC is dependent on transient mPTP-openings (low-conductance state; Hausenloy et al., 2010) and is defective in *CypD*-deficient mice (Lim et al., 2007). Interestingly, the ability to undergo IPC decreases in mice with age while treatment of IRI with CsA remains effective (Peart et al., 2014). Thus, the dual role of PaCYPD in the aging process of *P. anserina* exhibits many similarities to the roles of CYPD in mammalian systems.

Overall, in the current study, we integrated PaCYPD and mPT into the complex molecular network involved in cellular quality and lifespan control. It appears that under “normal” conditions autophagy, which is regulated by ROS and the contribution of PaCYPD-mediated mPT, increases during aging as a pro-survival response counteracting age-related accumulation of impairments. This process can experimentally be induced via challenging “healthy” strains by mild stress and leads to the beneficial hormetic effect. The adaptive capacity of this pro-survival system is however restricted. Passing threshold levels it leads to the activation of components of “type I” PCD (e.g., metacaspases) and to death of senescent cultures. Under special conditions of excessive, non-physiological stress the system can turn to a system in which no attempts to counteract molecular impairments are induced anymore (i.e., induction of beneficial levels of autophagy). Under these conditions it leads to “type II” cell death and a rapid deterioration.

## Author contributions

HO and PK designed the study. PK, AJ, and AH performed experiments and analyzed the data. HO and PK wrote the manuscript. HO supervised the study. All authors read the final version of the manuscript.

### Conflict of interest statement

The authors declare that the research was conducted in the absence of any commercial or financial relationships that could be construed as a potential conflict of interest.

## References

[B1] Baines C. P. Kaiser R. A. Purcell N. H. Blair N. S. Osinska H. Hambleton M. A.. (2005). Loss of cyclophilin D reveals a critical role for mitochondrial permeability transition in cell death. Nature 434, 658–662. 10.1038/nature03434 15800627

[B2] Bardiya N. Shiu P. K. (2007). Cyclosporin A-resistance based gene placement system for *Neurospora crassa*. Fungal Genet. Biol. 44, 307–314. 10.1016/j.fgb.2006.12.011 17320431

[B3] Basso E. Fante L. Fowlkes J. Petronilli V. Forte M. A. Bernardi P. (2005). Properties of the permeability transition pore in mitochondria devoid of cyclophilin D. J. Biol. Chem. 280, 18558–18561. 10.1074/jbc.C500089200 15792954

[B4] Bonora M. Wieckowski M. R. Chinopoulos C. Kepp O. Kroemer G. Galluzzi L.. (2015). Molecular mechanisms of cell death: central implication of ATP synthase in mitochondrial permeability transition. Oncogene 34, 1475–1486. 10.1038/onc.2014.96 24727893

[B5] Brust D. Daum B. Breunig C. Hamann A. Kühlbrandt W. Osiewacz H. D. (2010a). Cyclophilin D links programmed cell death and organismal aging in *Podospora anserina*. Aging Cell 9, 761–775. 10.1111/j.1474-9726.2010.00609.x 20626725

[B6] Brust D. Hamann A. Osiewacz H. D. (2010b). Deletion of *PaAif2* and *PaAmid2*, two genes encoding mitochondrial AIF-like oxidoreductases of *Podospora anserina*, leads to increased stress tolerance and lifespan extension. Curr. Genet. 56, 225–235. 10.1007/s00294-010-0295-1 20306265

[B7] Carreira R. S. Lee Y. Ghochani M. Gustafsson Å. B. Gottlieb R. A. (2010). Cyclophilin D is required for mitochondrial removal by autophagy in cardiac cells. Autophagy 6, 462–472. 10.4161/auto.6.4.11553 20364102 PMC3768271

[B8] Chen Y. Azad M. B. Gibson S. B. (2009). Superoxide is the major reactive oxygen species regulating autophagy. Cell Death Differ. 16, 1040–1052. 10.1038/cdd.2009.49 19407826

[B9] Cypser J. R. Johnson T. E. (2002). Multiple stressors in *Caenorhabditis elegans* induce stress hormesis and extended longevity. J. Gerontol. A Biol. Sci. Med. Sci. 57, B109–B114. 10.1093/gerona/57.3.B109 11867647

[B10] Daum B. Walter A. Horst A. Osiewacz H. D. Kühlbrandt W. (2013). Age-dependent dissociation of ATP synthase dimers and loss of inner-membrane cristae in mitochondria. Proc. Natl. Acad. Sci. U.S.A. 110, 15301–15306. 10.1073/pnas.1305462110 24006361 PMC3780843

[B11] Davalli P. Mitic T. Caporali A. Lauriola A. D'Arca D. (2016). ROS, cell senescence, and novel molecular mechanisms in aging and age-related diseases. Oxid. Med. Cell. Longev. 2016:3565127. 10.1155/2016/3565127 27247702 PMC4877482

[B12] Elmore S. P. Qian T. Grissom S. F. Lemasters J. J. (2001). The mitochondrial permeability transition initiates autophagy in rat hepatocytes. FASEB J. 15, 2286–2287. 10.1096/fj.01-0206fje 11511528

[B13] Elrod J. W. Molkentin J. D. (2013). Physiologic functions of cyclophilin D and the mitochondrial permeability transition pore. Circ. J. 77, 1111–1122. 10.1253/circj.CJ-13-0321 23538482 PMC6397958

[B14] Esser K. (1974). Podospora anserina, in Handbook of Genetics, ed King R.C. (New York, NY: Plenum Press), 531–551.

[B15] Galluzzi L. Vitale I. Abrams J. M. Alnemri E. S. Baehrecke E. H. Blagosklonny M. V.. (2012). Molecular definitions of cell death subroutines: recommendations of the Nomenclature Committee on Cell Death 2012. Cell Death Differ. 19, 107–120. 10.1038/cdd.2011.96 21760595 PMC3252826

[B16] Grimm C. Osiewacz H. D. (2015). Manganese rescues adverse effects on lifespan and development in *Podospora anserina* challenged by excess hydrogen peroxide. Exp. Gerontol. 63, 8–17. 10.1016/j.exger.2015.01.042 25616172

[B17] Groebe K. Krause F. Kunstmann B. Unterluggauer H. Reifschneider N. H. Scheckhuber C. Q.. (2007). Differential proteomic profiling of mitochondria from *Podospora anserina*, rat and human reveals distinct patterns of age-related oxidative changes. Exp. Gerontol. 42, 887–898. 10.1016/j.exger.2007.07.001 17689904

[B18] Hamann A. Brust D. Osiewacz H. D. (2007). Deletion of putative apoptosis factors leads to lifespan extension in the fungal ageing model *Podospora anserina*. Mol. Microbiol. 65, 948–958. 10.1111/j.1365-2958.2007.05839.x 17627766

[B19] Hamann A. Brust D. Osiewacz H. D. (2008). Apoptosis pathways in fungal growth, development and ageing. Trends Microbiol. 16, 276–283. 10.1016/j.tim.2008.03.003 18440231

[B20] Hamann A. Krause K. Werner A. Osiewacz H. D. (2005). A two-step protocol for efficient deletion of genes in the filamentous ascomycete *Podospora anserina*. Curr. Genet. 48, 270–275. 10.1007/s00294-005-0018-1 16160832

[B21] Hausenloy D. J. Lim S. Y. Ong S. G. Davidson S. M. Yellon D. M. (2010). Mitochondrial cyclophilin-D as a critical mediator of ischaemic preconditioning. Cardiovasc. Res. 88, 67–74. 10.1093/cvr/cvq113 20400621 PMC2936122

[B22] Izzo V. Bravo-San Pedro J. M. Sica V. Kroemer G. Galluzzi L. (2016). Mitochondrial permeability transition: new findings and persisting uncertainties. Trends Cell Biol. 26, 655–667. 10.1016/j.tcb.2016.04.006 27161573

[B23] Knuppertz L. Hamann A. Pampaloni F. Stelzer E. Osiewacz H. D. (2014). Identification of autophagy as a longevity-assurance mechanism in the aging model *Podospora anserina*. Autophagy 10, 822–834. 10.4161/auto.28148 24584154 PMC5119060

[B24] Kroemer G. El-Deiry W. S. Golstein P. Peter M. E. Vaux D. Vandenabeele P.. (2005). Classification of cell death: recommendations of the Nomenclature Committee on Cell Death. Cell Death Differ. 12(Suppl. 2), 1463–1467. 10.1038/sj.cdd.4401724 16247491

[B25] Kunstmann B. Osiewacz H. D. (2008). Over-expression of an S-adenosylmethionine-dependent methyltransferase leads to an extended lifespan of *Podospora anserina* without impairments in vital functions. Aging Cell 7, 651–662. 10.1111/j.1474-9726.2008.00412.x 18616635

[B26] Kunstmann B. Osiewacz H. D. (2009). The S-adenosylmethionine dependent O-methyltransferase PaMTH1: a longevity assurance factor protecting *Podospora anserina* against oxidative stress. Aging (Albany. NY). 1, 328–334. 10.18632/aging.100029 20157520 PMC2806012

[B27] Lecellier G. Silar P. (1994). Rapid methods for nucleic acids extraction from Petri dish-grown mycelia. Curr. Genet. 25, 122–123. 10.1007/BF00309536 8087879

[B28] Lim S. Y. Davidson S. M. Hausenloy D. J. Yellon D. M. (2007). Preconditioning and postconditioning: the essential role of the mitochondrial permeability transition pore. Cardiovasc. Res. 75, 530–535. 10.1016/j.cardiores.2007.04.022 17512507 PMC2080572

[B29] Linard D. Kandlbinder A. Degand H. Morsomme P. Dietz K. J. Knoops B. (2009). Redox characterization of human cyclophilin D: identification of a new mammalian mitochondrial redox sensor? Arch. Biochem. Biophys. 491, 39–45. 10.1016/j.abb.2009.09.002 19735641

[B30] Luce K. Osiewacz H. D. (2009). Increasing organismal healthspan by enhancing mitochondrial protein quality control. Nat. Cell Biol. 11, 852–858. 10.1038/ncb1893 19543272

[B31] Meiling-Wesse K. Barth H. Thumm M. (2002). Ccz1p/Aut11p/Cvt16p is essential for autophagy and the cvt pathway. FEBS Lett. 526, 71–76. 10.1016/S0014-5793(02)03119-8 12208507

[B32] Mesquita A. Weinberger M. Silva A. Sampaio-Marques B. Almeida B. Leão C.. (2010). Caloric restriction or catalase inactivation extends yeast chronological lifespan by inducing H_2_O_2_ and superoxide dismutase activity. Proc. Natl. Acad. Sci. U.S.A. 107, 15123–15128. 10.1073/pnas.1004432107 20696905 PMC2930563

[B33] Müller-Ohldach M. Brust D. Hamann A. Osiewacz H. D. (2011). Overexpression of *PaParp* encoding the poly(ADP-ribose) polymerase of *Podospora anserina* affects organismal aging. Mech. Ageing Dev. 132, 33–42. 10.1016/j.mad.2010.11.003 21145908

[B34] Nakagawa T. Shimizu S. Watanabe T. Yamaguchi O. Otsu K. Yamagata H.. (2005). Cyclophilin D-dependent mitochondrial permeability transition regulates some necrotic but not apoptotic cell death. Nature 434, 652–658. 10.1038/nature03317 15800626

[B35] Osiewacz H. D. Hamann A. Zintel S. (2013). Assessing organismal aging in the filamentous fungus *Podospora anserina*. Methods Mol. Biol. 965, 439–462. 10.1007/978-1-62703-239-1_29 23296676

[B36] Peart J. N. Pepe S. Reichelt M. E. Beckett N. See Hoe L. Ozberk V.. (2014). Dysfunctional survival-signaling and stress-intolerance in aged murine and human myocardium. Exp. Gerontol. 50, 72–81. 10.1016/j.exger.2013.11.015 24316036 PMC4096533

[B37] Philipp O. Hamann A. Servos J. Werner A. Koch I. Osiewacz H. D. (2013). A genome-wide longitudinal transcriptome analysis of the aging model *Podospora anserina*. PLoS ONE 8:e83109. 10.1371/annotation/03280dea-66ce-4ba6-8ac5-f985f51dea37 24376646 PMC3869774

[B38] Rizet G. (1953). [Impossibility of obtaining uninterrupted and unlimited multiplication of the ascomycete *Podospora anserina*]. C. R. Hebd. Seances Acad. Sci. 237, 838–840. 13107134

[B39] Rodriguez-Enriquez S. Kim I. Currin R. T. Lemasters J. J. (2006). Tracker dyes to probe mitochondrial autophagy (mitophagy) in rat hepatocytes. Autophagy 2, 39–46. 10.4161/auto.2229 16874071 PMC4007489

[B40] Rubinsztein D. C. Mariño G. Kroemer G. (2011). Autophagy and aging. Cell 146, 682–695. 10.1016/j.cell.2011.07.030 21884931

[B41] Sambrook J. Fritsch E. F. Maniatis T. (1989). Molecular Cloning: A Laboratory Manual. Cold Spring Harbour, NY: Cold Spring Harbour Press.

[B42] Schinzel A. C. Takeuchi O. Huang Z. Fisher J. K. Zhou Z. Rubens J.. (2005). Cyclophilin D is a component of mitochondrial permeability transition and mediates neuronal cell death after focal cerebral ischemia. Proc. Natl. Acad. Sci. U.S.A. 102, 12005–12010. 10.1073/pnas.0505294102 16103352 PMC1189333

[B43] Schneider M. D. (2005). Cyclophilin D: knocking on death's door. Sci. STKE 2005:pe26. 10.1126/stke.2872005pe26 15942033

[B44] Schweichel J. U. Merker H. J. (1973). The morphology of various types of cell death in prenatal tissues. Teratology 7, 253–266. 10.1002/tera.1420070306 4807128

[B45] Strobel I. Osiewacz H. D. (2013). Poly(ADP-ribose) polymerase is a substrate recognized by two metacaspases of *Podospora anserina*. Eukaryot Cell 12, 900–912. 10.1128/EC.00337-12 23584991 PMC3675990

[B46] Tropschug M. Barthelmess I. B. Neupert W. (1989). Sensitivity to cyclosporin A is mediated by cyclophilin in *Neurospora crassa* and *Saccharomyces cerevisiae*. Nature 342, 953–955. 10.1038/342953a0 2531848

[B47] Welter E. Thumm M. Krick R. (2010). Quantification of nonselective bulk autophagy in *S. cerevisiae* using Pgk1-GFP. Autophagy 6, 794–797. 10.4161/auto.6.6.12348 20523132

[B48] Yang W. Hekimi S. (2010). A mitochondrial superoxide signal triggers increased longevity in *Caenorhabditis elegans*. PLoS Biol. 8:e1000556. 10.1371/journal.pbio.1000556 21151885 PMC2998438

[B49] Zhao H. Joo S. Xie W. Ji X. (2013). Using hormetic strategies to improve ischemic preconditioning and postconditioning against stroke. Int. J. Physiol. Pathophysiol. Pharmacol. 5, 61–72. 23750305 PMC3669735

[B50] Zintel S. Schwitalla D. Luce K. Hamann A. Osiewacz H. D. (2010). Increasing mitochondrial superoxide dismutase abundance leads to impairments in protein quality control and ROS scavenging systems and to lifespan shortening. Exp. Gerontol. 45, 525–532. 10.1016/j.exger.2010.01.006 20080171

