# Supplementary material for: Cyclophilin D Is Involved in the Regulation of Autophagy and Affects the Lifespan of P. anserina in Response to Mitochondrial Oxidative Stress
Source: Front Genet. 2016 Sep 14;7:165. doi: 10.3389/fgene.2016.00165 (PMC5021683; doi:10.3389/fgene.2016.00165)
Supplement: Supplementary file 1 [file DataSheet1.docx]

Supplementary Material

Cyclophilin D is involved in the regulation of autophagy and affects the lifespan of *P. anserina* in response to oxidative stress

Piet Kramer, Alexander Thomas Jung , Andrea Hamann, Heinz Dieter Osiewacz^*^

*** Correspondence:** Prof. Dr. Heinz D. Osiewacz: osiewacz@bio.uni-frankfurt.de

**
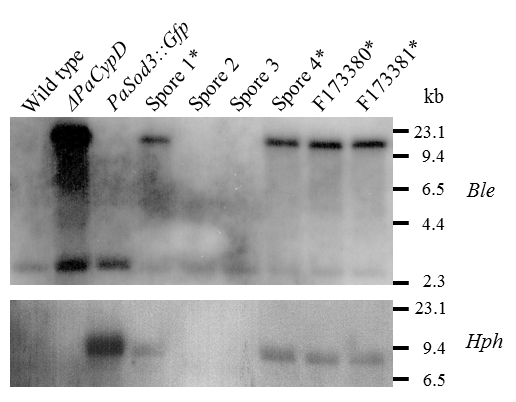
**

**Supplementary Figure 1.** Verification of *ΔPaCypD/PaSod3::Gfp* by Southern blot analysis of HindIII-digested genomic DNA from wild type, *ΔPaCypD*, *PaSod3::Gfp* and four dikaryotic offspring, two of which were sensitive (spore 2 and 3) and two of which were resistant (spore 1 and 4) to phleomycin and hygromycin B, isolated from a single recombined ascus of a cross between *ΔPaCypD* and *PaSod3::Gfp*. The spores 1 and 4 contain the concomitant deletion of *PaCypD* (associated with phleomycin resistance cassette) and the plasmid pPaSod3::gfp (carrying the phleomycin resistance cassette) from *PaSod3::Gfp* integrated in the genome, as indicated by the signals with probes against the phleomycin (*Ble*) and hygromycin (*Hph*) resistance genes. From self-crosses of these spores, monokaryotic offspring (F173380 and F173381) were isolated and used for subsequent experiments. The homokaryotic double mutants (*ΔPaCypD/PaSod3::Gfp*) are indicated by *.

**
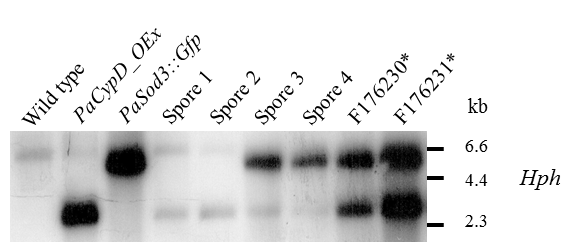
**

**Supplementary Figure 2.** Verification of *PaCypD_OEx/PaSod3::Gfp* by Southern blot analysis of EcoRV-digested genomic DNA from wild type, *PaCypD_OEx*, *PaSod3::Gfp* and four dikaryotic offspring, isolated from a single recombined ascus of a cross between *PaCypD_OEx* and *PaSod3::Gfp*. All four spores are heterokaryotic for the *PaCypD* overexpression cassette as indicated by the signals with probes against the hygromycin B resistance gene (*Hph*). One of two nuclei of the dicaryotic spores carries the *PaCypD* overexpression cassette plus hygromycin resistance cassette. Spore 3 and 4 are homokaryotic for the *PaSod3::Gfp* expression cassette plus hygromycin resistance gene (*Hph*, both nuclei of the dikaryotic spores carry the *PaSod3::Gfp* expression cassette), and thus contain the concomitant ectopic integration of pCypDEx1 from *PaCypD_OEx* and pPaSod3::gfp from *PaSod3::Gfp*. From self-crosses of these spores, monokaryotic offspring (F176230 and F176231) were isolated and used for subsequent experiments. The homokaryotic double mutants (*PaCypD_OEx/PaSod3::Gfp*) are indicated by *.

**
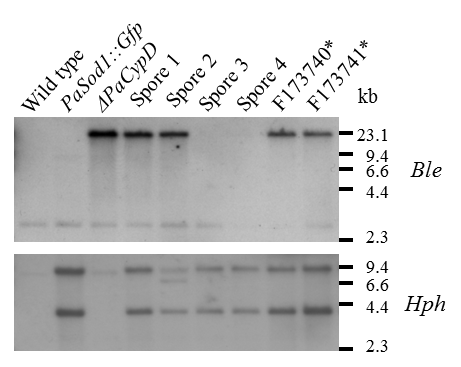
**

**Supplementary Figure 3.** Verification of *ΔPaCypD/PaSod1::Gfp* by Southern blot analysis of HindIII-digested genomic DNA from wild type, *PaSod1::Gfp*, *ΔPaCypD* and four dikaryotic offspring, isolated from a single recombined ascus of a cross between *PaSod1::Gfp* and *ΔPaCypD*. All four spores gave rise to mycelia resistant to hygromycin B, and thus are heterokaryotic for the hygromycin resistance gene (*Hph*, one of the two nuclei of the dicaryotic spores carries the *PaSod1::Gfp* expression cassette plus hygromycin resistance cassette). Two spores were phleomycin (*Ble*)-resistant (spore 1 and 2) and two were phleomycin-sensitive (spore 3 and 4). Spore 1 and 2 contain the concomitant deletion of *PaCypD* and the plasmid pPaSod1::gfp from *PaSod1::Gfp* integrated in the genome, as verified by the signals with probes against the phleomycin (*Ble*) and hygromycin (*Hph*) resistance genes. From selfcrosses of these spores, monokaryotic offspring (F173740 and F173741) were isolated and used for subsequent experiments. The homokaryotic double mutants (*ΔPaCypD/PaSod1::Gfp*) are indicated by *.

**
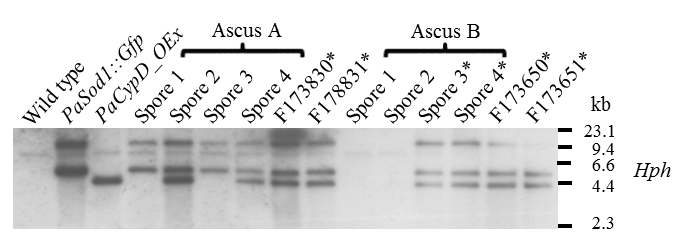
**

**Supplementary Figure 4.** Verification of *PaCypD_OEx/PaSod1::Gfp* by Southern blot analysis of EcoRV-digested genomic DNA from wild type, *PaSod1::Gfp*, *PaCypD_OEx* and eight dikaryotic offspring, isolated from two recombined asci (ascus A and B) of a cross between *PaSod1::Gfp* and *PaCypD_OEx*. All four spores of ascus A are heterokaryotic for the *PaSod1::Gfp* expression cassette, as shown by the signals with probes against the hygromycin B resistance gene (*Hph*, one of two nuclei of the dicaryotic spores carries the *PaSod1::Gfp* expression cassette plus hygromycin resistance cassette). Spore 1 and 2 of ascus B were recombined wild type strains and spore 3 and 4 were homokaryotic for the hygromycin resistance genes (*Hph*), and thus contained the concomitant ectopic integration of the plasmids pCypDEx1 from *PaCypD_OEx* and pPaSod3::gfp from *PaSod3::Gfp*. From selfcrosses of these spores, monokaryotic offspring (F173650 and F173651) were isolated and used for subsequent experiments. The homokaryotic double mutants (*PaCypD_OEx/PaSod1::Gfp*) are indicated by *.

**
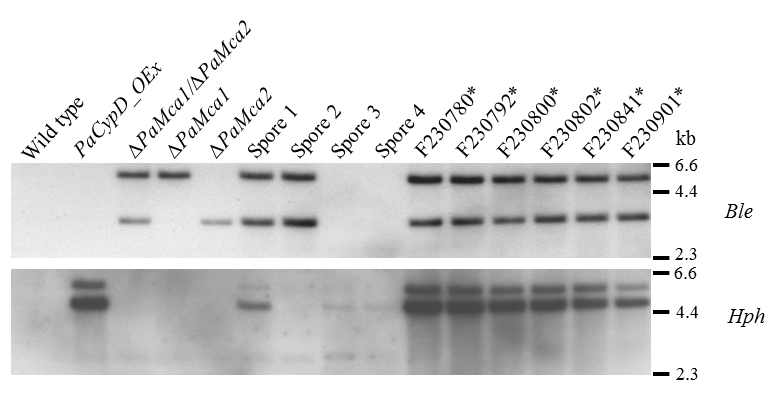
**

**Supplementary Figure 5.** Verification of *PaCypD_OEx/ΔPaMca1/ΔPaMca2 by* Southern blot analysis of HindIII-digested genomic DNA from wild type, *ΔPaMca1*, *ΔPaMca2*, the parental strains *PaCypD_OEx* and *ΔPaMca1/ΔPaMca2* and four dikaryotic offspring, isolated from a single recombined ascus of a cross between *PaCypD_OEx* and *ΔPaMca1/ΔPaMca2*. All four spores gave rise to mycelia resistant to hygromycin B, and thus are heterokaryotic for the hygromycin resistance gene (*Hph*, one of the two nuclei of the dicaryotic spores carries the *PaCypD* overexpression cassette plus hygromycin resistance cassette). Two spores were phleomycin (*Ble*)-resistant (spore 1 and 2) and two were phleomycin-sensitive (spore 3 and 4). Spore 1 contains the concomitant deletions of *PaMca1* and *PaMca2* and the ectopic integration of the plasmid pCypDEx1 from *PaCypD_OEx*, as verified by the signals with probes against the phleomycin (*Ble*) and hygromycin (*Hph*) resistance genes. From a selfcross of spore 1, monokaryotic offspring (F-numbers) were isolated and used for subsequent experiments. The homokaryotic triple mutants (*PaCypD_OEx/ΔPaMca1/ΔPaMca2*) are indicated by *.

**
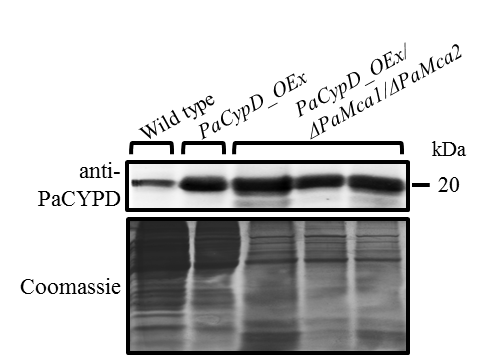
**

**Supplementary Figure 6.** Analyses of PaCYPD abundance in the triple mutant *PaCypD_OEx/ΔPaMca1/ΔPaMca2* in comparison to wild type and *PaCypD_OEx,* by western blot analysis. PaCYPD was detected by immunoblotting anti-PaCYPD in 100 µg total protein extracts. The Coomassie-stained gel is shown as loading control. The positions of molecular mass markers are indicated on the right.

**
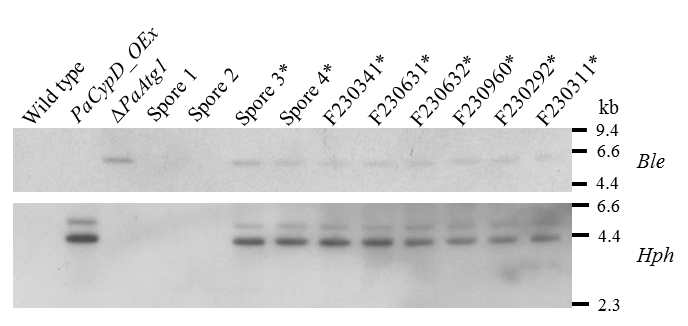
**

**Supplementary Figure 7.** Verification of *PaCypD_OEx/ΔPaAtg1* by Southern blot analysis of HindIII-digested genomic DNA from wild type, *PaCypD_OEx*, *ΔPaAtg1* and four dikaryotic offspring, two of which were sensitive (spore 1 and 2) and two of which were resistant (spore 3 and 4) to phleomycin and hygromycin B, isolated from a single recombined ascus of a cross between *PaCypD_OEx* and *ΔPaAtg1*. The spores 3 and 4 contain the concomitant deletion of *PaAtg1* and the ectopic integration of plasmid pCypDEx1 from *PaCypD_OEx*, as verified by the signals with probes against the phleomycin (*Ble*) and hygromycin (*Hph*) resistance genes. From selfcrosses of these spores, monokaryotic offspring (F-numbers) were isolated and used for subsequent experiments. The homokaryotic double mutants (*PaCypD_OEx/ΔPaAtg1*) are indicated by *.

**
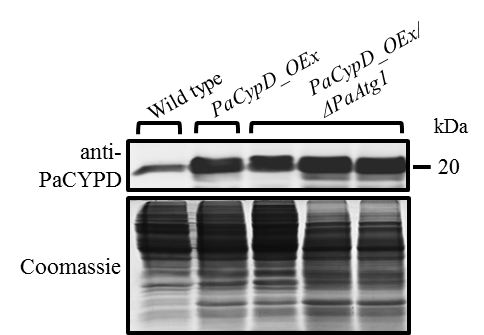
**

**Supplementary Figure 8.** Analyses of PaCYPD abundance in the double mutant *PaCypD_OEx/ΔPaAtg1* in comparison to the wild type and *PaCypD_OEx* by western blot analysis. PaCYPD was detected by immunoblotting anti-PaCYPD in 100 µg total protein extracts. The Coomassie-stained gel is shown as loading control. The positions of molecular mass markers are indicated on the right.


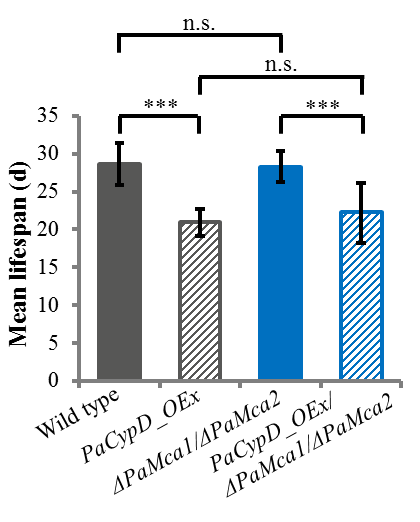


**Supplementary Figure 9.** Mean lifespan of 20 different isolates of the wild type, *PaCypD_OEx*, *ΔPaMca1/ΔPaMca2* and the triple mutant *PaCypD_OEx/ΔPaMca1/ΔPaMca2* on M2 medium. Data represent average ± SEM (2-tailed Wilcoxon rank-sum test) Not significant (n.s.), ***P < 0.001.


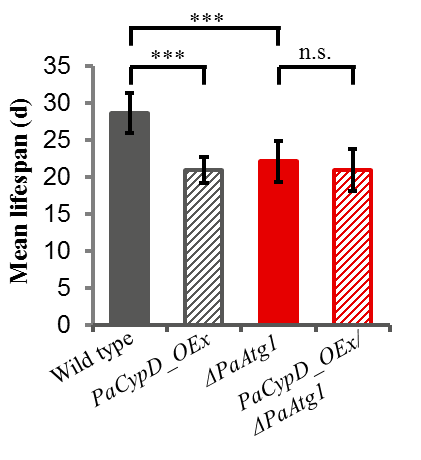


**Supplementary Figure 10.** Mean lifespan of wild type (n=20), *PaCypD_OEx* (n=20), *ΔPaAtg1* (n=16) and the double mutant *PaCypD_OEx/ΔPaAtg1* (n=15) on M2 medium. Data represent average ± SEM (2-tailed Wilcoxon rank-sum test) Not significant (n.s.), ***P < 0.001.


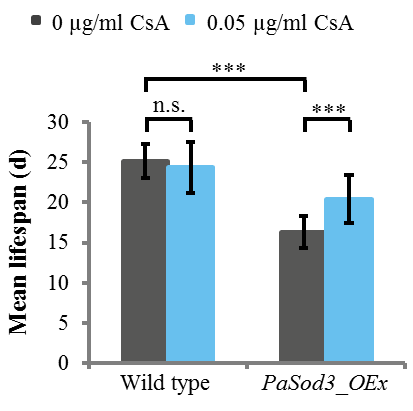


**Supplementary Figure 11.** Mean lifespan of wild type (n=33) and *PaSod3_OEx* (n=20) on M2 medium with 0 or 0.05 µg/ml cyclosporine A. Data represent average ± SEM (2-tailed Wilcoxon rank-sum test) Not significant (n.s.), ***P < 0.001.
